# Preparedness for practice, competency and skill development and learning in rural and remote clinical placements: A scoping review of the perspective and experience of health students

**DOI:** 10.1007/s10459-024-10378-4

**Published:** 2024-09-30

**Authors:** Stevie-Jae Hepburn, Syadani Riyad Fatema, Rikki Jones, Kylie Rice, Kim Usher, Jen Williams

**Affiliations:** Faculty of Medicine and Health, University of New England, Armidale, NSW 2350 Australia

**Keywords:** Rural health, Student experience, Competency based education, Preparedness for practice, Health education

## Abstract

When considering health professionals’ requirements and responsibilities, competence is a complex concept that extends beyond knowledge to encompass understanding, application, technical skills, problem-solving, and clinical judgment. Rural clinical placements provide a rich learning environment for students to improve their competencies, self-esteem, and preparedness for practice (PFP). This scoping review aimed to identify pre-registration health students’ perspectives on rural placement regarding competency and skill development, and enablers and barriers to learning. The methodology outlined by the Joanna Briggs Institute was followed. Out of 1186 records (366 duplicates), 821 were screened by title and abstract, 59 underwent full-text screening and 18 studies were included. Over half the studies included medicine students (*n* = 11). Reported competencies predominantly focused on self-assessed confidence regarding clinical skills. The reported barriers and enablers to learning highlighted the complexity of the placement experience and the importance of interpersonal factors, learner engagement and the supervisor’s role. The review identified a gap in the literature pertaining to student perspectives of competencies and skills not assessed or experienced as well as a lack of standardised measures for PFP. The identified enablers and barriers provide suggestions for rural placement design and emphasise the importance of the student learning experience. Future research could include multiple health disciplines and specific methodologies to identify health students’ perspectives regarding the development of competency, relatedness and autonomy, that is how to support health students to think, feel and act like health professionals.

**Supplementary Information:**

The online version contains supplementary material available at 10.1007/s10459-024-10378-4.

## Introduction

The importance of community-based education for health professionals has been long recognised to improve the health of communities by providing health professionals with an understanding of how local cultural beliefs and environmental factors can perpetuate health concerns (Cuff, 2014). Educators, health students and community members are all actively engaged in educational activities that are determined by the health needs of the community (World Health Organization, 1987). Community-based education provides an opportunity to broaden health students’ perspectives, career choices and readiness to work in rural environments (Fritsma et al., 2023; Kamien, 1975; Rural Health Workforce Australia, 2012). Placing students in rural and remote settings exposes them to the unique skills and experiences required to practice in these communities (Held et al., 2019). This community-based educational experience can increase students’ awareness of health professionals’ vital role in rural communities (Cuff, 2014; Kamien, 1975).

Previous research has indicated that rural placement experiences may significantly impact the intent to practice in rural contexts (Daly et al., 2013; Eley et al., 2012; Kelly et al., 2022; King et al., 2016; Taylor & Glass, 2018). Understanding the experiences of health students may provide valuable insight into their learning and, consequently, inform future program development to target student learning needs. The global healthcare worker shortage is well documented, with the WHO reporting 17 physicians per 10,000 individuals globally. Moreover, health professionals are unevenly distributed across most Organisation for Economic Cooperation and Development (OECD) countries. For example, 39.8 doctors per 10,000 people in Australia, 24.6 in Canada, 31.7 in the United Kingdom and 9.3 in Thailand and 1.89 in Zimbabwe (WHO, 2024a, b). There is a consistent disparity between rural and urban contexts across all OECD countries; for example, the average across OECD countries is 4.3 physicians per 1000 people in urban contexts and 2.8 in rural contexts. In Australia, for instance, there are 132,000 clinicians presently working in regional, rural, and remote areas across Australia, compared to 386,000 clinicians in metropolitan areas (Australian Institute of Health & Welfare, 2022).

The OECD has forecast that population aging, rising incomes and technological advances will continue to boost the demand for healthcare workers. Efforts are needed to increase the attraction and retention of healthcare workers (OECD, 2023), particularly those in rural areas. Similarly, the United Nation’s Sustainable Development Goals include the training, recruitment and retention of healthcare workers as key to good health and wellbeing, particularly through improved access to health services (Goal 3.C) (United Nations, 2015). In Australia, there is a need to facilitate rural placement experiences due to the shortage of rural health professionals (Smith et al., 2018), and to help recruit emerging practitioners to these areas (McBride et al., 2015).

Across all contexts, health professionals work to safeguard human health by using evidence-based medicine and care practice (WHO, 2013) whilst adhering to regulatory and professional standards and competencies. When considering health professionals’ requirements and responsibilities, competence is a complex concept that extends beyond knowledge to encompass understanding, application of knowledge, technical skills, problem-solving, and clinical judgment. Competencies include skills, knowledge, values, and attitudes essential to a specific health profession, and are embedded in health discipline standards for health care, providing consistency for health students, clinicians, educators and patients/clients (Verma et al., 2006). Competency is defined in the Medical Subject Headings (MeSH) as “the capability to perform acceptably those duties directly related to patient care” (National Library of Medicine, 2024). Similarly, the Australia Health Practitioner Regulatory Agency (Ahpra) outlines that “practice” includes “any role, whether remunerated or not, in which the individual uses their skills and knowledge as a practitioner in their regulated health profession” (Ahpra, 2022, p. 30). Given the complexity of health professionals’ roles, becoming a practitioner requires more than simply meeting competencies and demonstrating skills. It involves a complex process that includes acquiring attitudes, behaviours, and values (Mylrea et al., 2017). That is, it is through internalisation that a health student begins to think, feel, and act like a health professional (Cruess et al., 2019).

The MeSH defines competency-based education as “educational programs designed to ensure that students attain prespecified levels of competence in a given field or training activity” (National Library of Medicine, 2024). Clinical placements are included in educational programs and are crucial in supporting students’ preparedness and readiness as competent and responsive future health professionals (Brennan et al., 2010). The placement environment provides numerous learning opportunities for students, such as experiential learning, where learners continuously test their preconceived ideas and understanding and integrate new concepts (Kolb, 2015). Through these experiential learning opportunities in various health-related contexts, students are prepared for clinical duties and develop effective communication skills to work with patients and colleagues (Morrow et al., 2012). In addition to helping students improve their level of competency, clinical placement settings also help students build their professional identity, self-esteem, organisational abilities, and practice readiness (O’Doherty et al., 2021). In particular, self-efficacy is regarded as an appropriate measure of an individual’s beliefs in their skills and abilities to complete a task (Bandura, 1997) and is a measure of progress in clinical education (Mullen et al., 2015).

Preparedness for practice (PFP) and health profession education are key considerations in workforce retention (Brennan et al., 2024). PFP is influenced by a health professional’s professional identity and their understanding of professionalism within their health discipline. It has been suggested that professional identity is an important educational objective whereby health students are prepared to think, feel and act like health professionals (Curess et al., 2019). Strong professional identity improves confidence and competence, which in turn supports health professionals’ PFP (e.g., management of clinical situations and decision-making) (Qin et al., 2024).

It is surprising that PFP is not clearly defined in the literature even though there has been a significant increase in research in this area (Monrouxe et al., 2016; Burnford et al., 2014). A rapid review by Monrouxe et al. (2014; *n* = 81 articles) called for a clear definition of PFP and the inclusion of multiple stakeholder perspectives to understand the requirements and needs of junior doctors. More recently, a systematic review (Brennan et al., 2024; *n* = 14 articles) explored PFP for allied health professionals within the United Kingdom and reported there was a paucity of medium or high-quality research on PFP. Similarly, Aggarwall et al., (2023) echoed the importance of clearly defining PFP with reference to competency-based medical education. The rapid review (*n* = 34 articles) and focus groups with early-career family physicians (*n* = 59) indicated there was inconsistency in the literature regarding PFP. Conceptual clarity was even absent when comparing studies from the same discipline. The findings from Aggarwall et al., however, provided four themes for the conceptualisation of PFP: competence, capability, adaptability and self-confidence (i.e. self-efficacy and self-concept) (Aggarwall et al., 2023). PFP defined in these terms, was used to guide the approach in the present review.

Often, student voice is represented as a source of data rather than as a means of positioning students as partners in the learning process (Barradell & Bell, 2021). The aim of this review is to explore the literature regarding student perspectives on their learning experience with regard to competency and skill development and enablers and barriers to learning. The findings may inform future program development *and* future research that specifically captures and highlights student perspectives.

The OECD has provided a typology to classify rural and remote communities (see Brezzi et al., 2011) however, rural health is not consistently defined in the literature. Concerning health care services, demographic factors (e.g. population density and size), regional economic factors (e.g., distance to advanced health services), and cultural barriers are some of the characteristics that are used to define rural contexts (Rusaanes et al., 2024; Versace et al., 2021). For example, medium rural areas are defined as being in or within 10-km (6.21 miles) of a town with a population between 5000 and 15,000 (Versace et al., 2021). For the purpose of this review, the term ‘rural’ is synonymous with ‘remote’, ‘isolated’ and ‘decentralised’.

### Theoretical perspective

Healthcare systems and institutions are complex environments, and providing care involves dynamic interactions between multiple stakeholders (e.g. patients/clients, family members, clinicians, health students, and clinical support staff). Bronfenbrenner’s Ecological Systems Theory (EST; 1979) provides a lens through which we can examine the experience of students in complex healthcare environments. Bronfenbrenner’s model has been applied in education and social science to examine the interactions between educators, students, and the learning environment, in health promotion programs (e.g., Kok et al., 2008), rural clinical placement (e.g., (Killam & Carter, 2010)), and interprofessional education and interprofessional collaborative practice (e.g., D’Amour & Oandasan, 2005; Oandasan & Reeves, 2005). EST has also been applied to the development of competency frameworks in healthcare (e.g., Batt et al., 2021). Recently, Bronfenbrenner’s EST has been used to evaluate medical students’ experiences of equality and inclusion in the pre-clinical and clinical learning environment (Nolan & Owen, 2024).

As D’Amour and Oandasan (2005) outlined, multiple factors influence health professional education programs within the educational and professional systems. Within an education system, meso-level (institutional) factors include resources, leadership, and administrative processes, among others. Micro-level (individual) factors include teaching practices, the learning context, and key stakeholders. The education system operates in connection with the professional system; for example, when health students attend placement, they move through a process where their experience within the educational system influences and shapes their progress and development as they move through the professional system. The professional system similarly includes meso-level factors, such as organisational structures and governance, and interactional factors at the micro level, such as a sense of belonging, shared vision among staff and interprofessional collaboration (Oandasan & Reeves, 2005). The learner is at the centre of the educational system, whereas the patient/client is the centre of the professional system (D’Amour & Oandasan, 2005). In the context of this present review, we are focusing on learning within the professional environment.

Given the importance of learner engagement (e.g. self-efficacy and confidence), professional identity and PFP, Self-determination Theory (SDT; Deci & Ryan, 1985) provides an additional theory through which we can further explore student experience and learning. The main goal of this review is to highlight and foreground the reported learning experiences of students, that is, the student’s perspective regarding their learning in the rural context. As educators, it is imperative that we consider the learners’ needs, if they feel supported in the learning environment and how we can support their motivation and engagement. SDT posits that intrinsic motivation is self-determined and bolstered by three psychological needs: autonomy, relatedness, and competence. Autonomy refers to feeling control over behaviours and goals and the ability to act. Competence involves mastering tasks and learning new skills, and relatedness encompasses a sense of belonging, connection to others and attachment. According to Deci and Ryan (1985; 2001) a cooperative learning environment can support intrinsic motivation, create a positive emotional tone, and increase learner engagement. In addition, acquiring knowledge with the intent of application (i.e. putting it to use, contextualised learning) increases intrinsic motivation, consequently improving the quality of learning. Both theories are highly relevant to the present review as they provide a means to explore the reported enablers and barriers to learning from the perspective of health students on rural placement.

In summary, the placement environment is influenced by multiple factors and shapes the learner’s experience. In addition, the learner is an active agent in their learning experience, influenced by their self-determination, among other influences, such as life experiences and cultural background. A health student’s professional identity is linked to and shaped by their learning experience on placement and the placement learning environment (Daly et al., 2013; D’Amour & Oandasan, 2005; Tan et al., 2017). Deeper community engagement and strong connections can support health students’ professional identity as rural health professionals, which increases commitment to rural communities and the likelihood of health professionals practising in rural contexts once registered (Brownlee et al., 2015; Owens et al., 2018).

### Rationale

Previous reviews have focused on quality and features in rural placement (Green et al., 2022), stakeholder perspective and experience of rural placement (Somporn et al., 2018), challenges experienced by students attending placement (Killam & Carter, 2010), student experience when failing on placement (Milgate et al., 2024), and institutional support structures for students (Rogers, 2021). The present review aims to identify trends in the literature, explore the reported perspective of learners in rural placement settings, identify and map health students’ lived experience and perspective of their PFP and competency/skill development in the rural placement learning environment. The learning context includes the clinical placement environment, which is defined as an environment that provides healthcare services to the public or patients (General Medical Council, 2024). When students attend a rural community for placement, they are immersed in the community. Therefore, the learning environment extends to both formal and informal contexts.

Identifying enablers and barriers to learning may inform future training and program development. The aim is to consider and highlight the student voice (Milgate et al., 2024) and emphasise the importance of identifying learner-identified enablers and barriers that support learning and skill development. A scoping review was considered suitable for exploring the literature surrounding the experience of health students on placement in remote rural areas because it provides the opportunity to conduct a broad review, including diverse sources on the topic (Pollock et al., 2021).

### Aims

The scoping review aimed to identify the perspective of pre-registration health students on rural placement regarding competency and skill development, as well as enablers and barriers to learning. International literature sought to create a global perspective on this topic and the following research questions were proposed:

1. What is the reported impact of rural placement on preparedness for practice, competency and skill development from the perspective of health students?
2. What are the reported competencies and skills that health students believe are effectively taught, practised, and assessed in rural clinical learning environments?
3. What are the reported enablers and barriers to learning from the perspective of pre-registration health students on rural placement?

## Method

### Design

A scoping review design was undertaken following the methodology outlined by Peters et al., (2020) from the Joanna Briggs Institute (JBI). In addition, the framework from Arksey and O’Malley (2005) and the PRISMA-ScR checklist guided the stages of the review (Tricco et al., 2018). The research team were situated within the School of Health at an Australian University and included academics with diverse professional experience in the disciplines of psychology, nursing, medicine and education. Reflexivity was used throughout the review, whereby the research team acknowledged their professional experience and perspectives, particularly when engaging in data extraction and analysis.

### Search strategy

A literature review protocol was developed in consultation with a health librarian. The protocol outlined the format for eligibility, and was aligned with the PCC mnemonic, *population*, *concept* and *context* (Pollock et al., 2021). The *population* (participants) were defined as students enrolled in undergraduate or pre-registration health profession programs. No limitations were applied to the health disciplines included. The *concept* of interest included students’ perceptions regarding competency development, preparedness, and readiness for practice after completing clinical placement and how this concept was represented in empirical, peer-reviewed studies that could be used to inform the development of future research projects. Therefore, non-peer-reviewed and grey literature were not included. The *context* was defined as clinical placement within a rural or remote setting. Due to the review’s exploratory nature, the search strategy’s development involved an iterative process resulting in multiple preliminary searches to identify keywords and concepts relevant to the review aims and objectives. The key terms, titles and abstracts of relevant articles were analysed to refine the search strategy further. Following this, the author (SH) consulted the university Health librarian to identify relevant keywords and refine the strategy based on the eligibility criteria (see Table 1). Theoretical papers that informed placement design were excluded, as were studies that investigated the career trajectory of students. The search strategy was piloted in Web of Science and CINAHL to ensure relevant articles were identified, and the strategy was further refined.

**Table 1:** Eligibility criteria

	Inclusion	Exclusion
Type of evidence	All study types reported in English	Editorials, newspaper articles, magazines, opinion papers
	Peer-reviewed conference abstracts	Non-peer reviewed, conference abstracts, discussion papers
	Peer-reviewed publications	Dissertations / theses
	Empirical (primary) studies	Secondary (review) studies
Population	Undergraduate / pre-registration	Post-registration health professionals
	All health disciplines	
Concept	Professional competency	Career trajectory after placement
	Skill development	Models for placement design
	Self-perceived competence and confidence	Attitudes towards rural contexts and rural practice
	Student perception, experience and feedback	Remote learning experiences, online coursework
	Readiness and preparedness	
Context	All clinical placement	Continuing professional training
	Rural and remote context	Simulated, tele-simulated
	Traditional (in-person)	

#### Search and screening process

Two reviewers (SH, RJ) independently completed the searches using all key terms across the databases: EBSCOhost (including CINAHL), InformIT, ProQuest PsycInfo, Scopus, MEDLINE, Web of Science, and Embase (search completed 18th and 19th of December 2023- full details provided in the Supplementary file). Any discrepancies were discussed, and consensus was reached before a final search (21st December 2023), and the records were exported to EndNote reference management software (Clarivate, 2023). Editorial and non-peer-reviewed sources were excluded, and no date range was applied to the search. The records were exported from EndNote to Covidence management software (Clarivate, 2020), and duplicates were removed. The sources included were peer-reviewed publications reported in English. Hand-searching of individual journals was completed and yielded one article; however, it was excluded from the full-text review.

Two researchers (SH, SRF) independently screened the records’ title and abstracts against the eligibility criteria (Table 1) with high inter-rater reliability (k = 0.90) (McHugh, 2012), and proportionate agreement of 98%. Two researchers (SH, SRF) completed the full-text screen, and each source was independently assessed against the inclusion and exclusion criteria (inter-rater reliability k = 0.78) and proportionate agreement was 89% (calculated in Covidence).

#### Data extraction and synthesis

The data extraction was guided by the JBI manual (Aromataris & Munn, 2020) and the recommendations of Pollock et al., (2022). The review objective, research questions, and theoretical frameworks were used to create the data extraction tool which was piloted by one author and refinements were made and discussed with a second author. One researcher completed the extraction (SH) the second researcher cross-checked (SRF), and both researchers engaged in discussion during the charting process. Article quality was appraised using the Mixed Methods Assessment Tool (MMAT) and the overall MMAT score was calculated and reported per the author’s guidance (Hong et al., 2018). As per the guidance from Pollock et al., (2022), articles were not excluded based on quality appraisal and the scores were reported in the results.

To address Research Question One, the findings from each study were recorded in categories relating to PFP, competencies and skills. For instance, PFP is not clearly defined in the literature (Aggarwal & Abdelhalim, 2023). The rapid review by Aggarwal and Abdelhalim (2023) provided an outline of the constructs of PFP that were used to guide the results of this review. References to PFP in the included articles were summarised as headings in Table 5, such as autonomy, confidence, professional knowledge, identity and belonging, and self-efficacy. Similarly, only the competencies and skills reported in the included studies have been included in the results. Research Question Two focused on student perception regarding competencies effectively taught, which was included in the data extraction chart.

As per the guidance from Pollock et al., (2022), a simple thematic analysis was completed to address Research Question Three. A deductive approach allowed for the reported barriers and enablers to be identified according to an established theoretical framework, Ecological Systems Theory (EST; Bronfenbrenner, 1979). The framework was selected prior to the data charting process; therefore, the data could be extracted as per the proposed research question. One author completed the analysis (SH), which involved unitisation within the articles, collating codes into the data extraction chart (crossed-checked by a second author, SRF), the development of categories (grouped codes) and themes (Braun & Clarke, 2021; Erlingsson & Brysiewicz, 2017). The themes aligned with the influential factors at each level in EST and are displayed in a mind map and a narrative summary with reference to the relevant articles. The mind map was created with a graphic organiser program: Coggle (www.coggle.it).

## Results

**Fig. 1:**
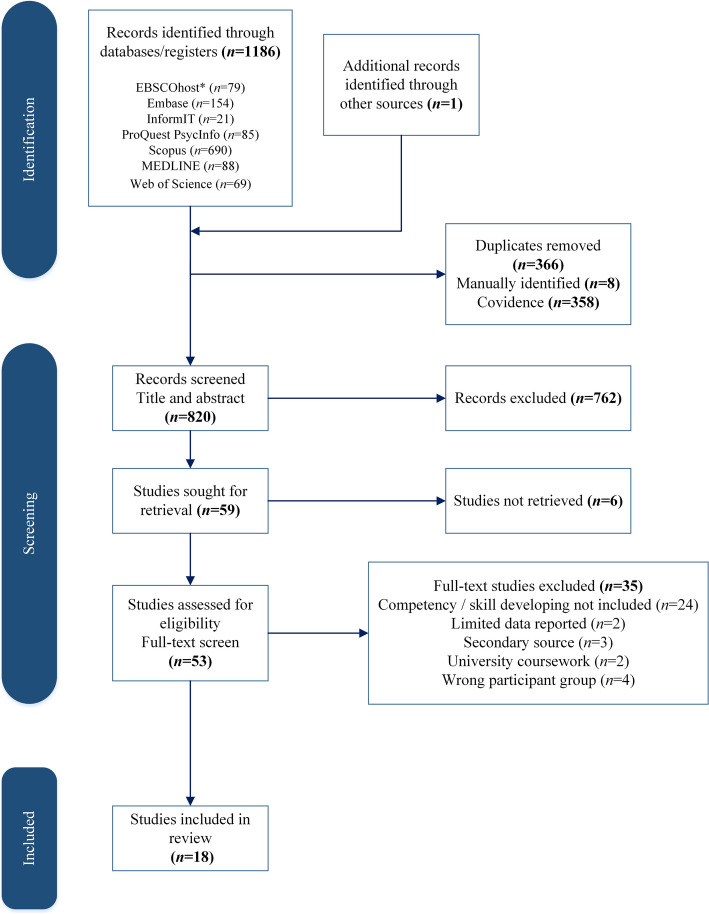
PRISMA Study selection *Embase Complete, Source Complete, CINAHL Complete, EconLit, Education Source, GreenFILE, Health Business Elite, Health Source—Consumer Edition, Health Source: Nursing/Academic Edition, Humanities Source, Library, Information Science & Technology Abstracts, MAS Ultra—School Edition, MasterFILE Complete, Newspaper Source Plus, Newswires, Psychology and Behavioral Sciences Collection, Regional Business News, Religion and Philosophy Collection, SPORTDiscus with Full Text, The Serials Directory, MasterFILE Premier Reference eBook Subscription (EBSCOhost), MAS Reference eBook Collection, MLA Directory of Periodicals, MLA International Bibliography with Full Text

The total records (*n* = 1186) were entered into Covidence (Clarivate, 2020) and duplicates were removed (*n* = 366). A total of 820 sources were included in the title and abstract screen. Fifty-nine sources met the inclusion criteria for full-text screening and were sought for retrieval. Six studies were unable to be retrieved from the library catalogue. Studies were excluded due to insufficient results, methodological limitations, or failure to meet the primary variable of the review (exclusion reasons are included in Fig. 1). Data extraction was completed on 18 studies.

No geographic limitations were applied to the search strategy to ensure a global perspective on the topic. However, the majority of studies were from Australia (*n* = 13), with limited representation from other countries (see Table 2). Fifty per cent of the included studies included quantitative methodologies (e.g., pre- and post-placement surveys).

**Table 2:** Study characteristics of total included studies (N = 18)

Study characteristics	Summary
Range	2004–2023
Country	USA^*n*=1^, Australia^*n*=13^, Japan^*n*=1^, New Zealand^*n*=1^, Scotland^*n*=1^, Uganda^*n*=1^
Methods	Focus groups^*n*=2^, interviews^*n*=2^, survey^*n*=7^, quasi-experiential^*n*=2^, Multi or mixed-methods^*n*=5^
Population	Students only^*n*=15^, students and clinical supervisors^*n*=2^, students, clinical supervisors* and patients/clients^*n*=1^

*Clinical supervisors: general practitioners, clinical educators, preceptors, mentors. Number superscript identifies number of articles

To allow for comparison across study designs, the included studies were grouped and presented as per the study design (e.g. quantitative, qualitative, and mixed or multi-methods study designs). The quantitative studies (*n* = 9) (Table 3) included study-specific instruments or measures (Edwards et al., 2004; Graham et al., 2023; Hunsaker et al., 2006), validated measures (McLean et al., 2010; Rudland et al., 2011; Witney et al., 2018) and only one study (McNair et al., 2005) included an adapted version of Kirkpatrick’s Model of evaluation (Barr et al., 2000). The multi or mixed-methods study designs (Table 4) included sequential designs, for example, focus groups to inform a cross-sectional survey (Lyon et al., 2008) or a study-specific pre- and post-placement survey followed by a post-placement interview or focus group (Bennett et al., 2013; Ohta et al., 2019; Young et al., 2008). One study included a pre- and post-placement survey, research project task, and open-ended feedback (Young et al., 2007). The qualitative study designs (*n* = 4) reported the student perspective through focus groups (Aggar et al., 2020; Furness et al., 2020), semi-structured interviews (Daly et al., 2013) and multiple datasets (e.g. reflective journals, semi-structured interviews) (Gupta & Howden, 2022) (Table 5).

**Table 3:** Characteristics of studies—quantitative study design

References	Title	Aims/objectives	Study design/methodsMeasures	Population	Key findings	MMAT Score
Edwards et al. (2004) Australia	The impact of clinical placement location on nursing students’ competence and preparedness for practice	Examine changes in students’ satisfaction with, competence, confidence, and organisation for clinical practice before and after a final year clinical practicum (across time) and between metropolitan and rural cohorts	Quasi-experimental—pre- and post-survey (study-specific) 5-point Likert agreement—confidence, competence and organisation for placement 12 items—clinical issues, 5-point scale of importance	Students: Nursing (pre-test *n* = 137, post-test *n* = 121) Final year	Students in both contexts reported higher confidence, competence and organisation post-placement No reported differences between contexts	***
Graham et al. (2023) Australia	Australian rural medical students’ perceived readiness for work as a junior doctor: A cross-sectional national survey	To report self-perceived readiness for work as a junior doctor in a national cohort of rural clinical school students	Cross-sectional survey (study-specific) Readiness as a junior doctor—5-point Likert agreement Readiness for intern tasks—14 items, 10-point scale Self-perceived support, 5-point Likert agreement	Students: Medicine (*n* = 668) 17 medical schools	86% of students felt rural clinical school (RCS)placement had prepared them for practice, however, self-rated readiness for intern tasks varied Ethical and professional dilemmas were rated lower	****
Hunsaker et al, (2006) USA	Medical students’ assessment of skill development in rural primary care clinics	To assess students’ self-perceptions of their ability to provide care (acute, chronic, preventative), communicate with patients and understand the community	Pre- and post-survey (study specific) Self-assessed skills across 11 dimensions (97 items) 6-point Likert confidence rating	Students: Medicine (*n* = 96)	A significant increase in confidence across all 97 self-assessed skills Highly assessed skills were chronic problem management, patient education and maintenance, and undifferentiated and acute problems Highest ranked skill gains were in understanding of healthcare systems and community	***
McLean et al. (2010) Australia	A multi-university evaluation of the rural clinical school experience of Australian medical students	To evaluate the experiences of medical students who attended rural clinical schools during 2006, using the rural-specific questionnaire	Cross-sectional survey Rural Clinical School Evaluation 2006 (DeWitt et al., 2005) 6-point Likert, 29 items—8 general experience, 4 skill development Clinical supervision 15 items Free-text comments	Students: Medicine (*n* = 125) 6 universities	Students reported agreement above 80% on general experience and skills development in the RCS. They also shared positive feedback regarding the quality of teaching experiences and the opportunities for varied learning experiences	**
McNair et al. (2005) Australia	Australian evidence for interprofessional education contributing to effective teamwork preparation and interest in rural practice	To outline the educational model and the findings from student evaluations	Quasi-experiential pre- and post-survey (study specific) Modified Kirkpatrick’s model of evaluation. Agreement 5-point Likert Competencies relevant to teamwork and collaboration (31 items pre- and 52 items post) 12-month follow-up survey—18 items Likert agreement and 4 open-ended questions	Students: Medicine (*n* = 39) Nursing (*n* = 38) Physiotherapy (*n* = 5) Pharmacy (*n* = 6) 48% rural campus	The Rural Interprofessional Education (RIPE) supported the development of interprofessional knowledge, beliefs, and attitudes. It increased self-reported confidence in engaging in interprofessional practice and improved teamwork skills	****
Rudland et al. (2011) New Zealand	The clinical skills experience of rural immersion medical students and traditional hospital placement students: A student perspective	To compare the self-reported experience and self-perceived competence in performing clinical skills between fifth-year students in rural and urban hospitals	Confidence in Common Procedures and Conditions Survey (Spike & Veitch 1991) Self-perceived confidence and experience ratings—4-point ordinal scale	Students: Medicine Rural (*n* = 6) Urban (*n* = 17)	Rural placement students reported greater experience with examinations and patient education, whereas urban students reported greater confidence and experience in interpretative and investigative skills	***
Wakida et al. (2015) Uganda	Health-profession students’ teaching and learning expectations in Ugandan medical schools: Pre- and post-community placement comparison	To assess the teaching and learning expectations before and after placement of health-profession students in community-based programs	Cross-sectional survey Structured and unstructured questions. Examples questions (nd.) Focus: What did students expect to learn on placement? How would their lives be affected? What are the minimum competencies?	Students: pre (*n* = 454), post (*n* = 305) Pharmacy Laboratory science Nursing Medicine 1st—4th year	Students ranked learning expectations as interpersonal skills, community engagement, community diagnosis, lifestyle practices, clinical skills, and patient management Peer-to-peer mentoring was promoted through the inclusion of students from different health programs	***
Webster et al. (2010) Australia	Undergraduate nursing students’ experiences in a rural clinical placement	Identify aspects of rural placements in Aboriginal communities that were effective in engaging nursing students in the learning process	Pre- and post-placement 10 items adapted from Australian Catholic University fieldwork evaluation form 5-point Likert agreement scale	Students: Nursing (*n* = 8)	Students participated in contextualised learning experiences and developed an awareness of and knowledge about Aboriginal health and cross-cultural issues Increased confidence and sense of achievement and independence	***
Witney et al. (2018) Australia	Block versus longitudinal integrated clerkships: Students’ views of rural clinical supervision	To compare the reported experience of clinical supervision and self-rated competence of students in rural Longitudinal Integrated Clerkship(LIC) and rural block rotation	Australian Rural Clinical School Exit Survey (FRAME questionnaire) Clinical supervision—14 statements rated on 5-point Likert agreement scale Overall satisfaction Self-rated clinical competence 5-point Likert scale for 8 statements related to confidence	Students: LIC (*n* = 279) Block rotation (n = 168) 13 medical schools	No reported difference between the supervisor scores for clinical supervisors or self-rated clinical confidence. The findings suggested the importance of the student-supervisor relationship and the benefits of the rural context itself as a positive factor in clinical education	****

*MMAT scoring: 100% quality criteria met = *****; 80% = ****; 60% = ***; 40% = **; 20% = *

**Table 4:** Characteristics of included studies—Mixed or multi-methods study design

References	Title	Aims/objectives	Study design/methods Measures	Population	Key findings	MMAT Score
Bennett et al. (2013) Australia	Supporting rural/remote primary health care placement experiences increases undergraduate nurse confidence	Describe the impact of a structured and comprehensive educational, clinical placement experience on undergraduate nurses’ levels of confidence in the areas of primary health care and culturally knowledgeable practice	Pre- and post-survey (study-specific): Baseline—perceptions of rural life, Indigenous health and primary health care. Confidence logs—5-point Likert Focus group—questions not defined.Follow-up 3-month phone interview	Students: Nursing (*n* = 31) Two universities	Increased self-assessed confidence across all domains (interactions with Indigenous peoples) Positive impact on knowledge, skills and attitudes towards rural health Immersion provides authentic learning experiences	***
Lyon et al. (2008) Australia	Students’ perceptions of clinical attachments across rural and metropolitan settings,	To examine students’ perceptions of key features that facilitate their learning in clinical practice environments and to compare their perceptions across rural and metropolitan settings	Focus group—questions not defined Survey informed by focus group. Behavioural 5-point Likert Topics: clinical teaching, opportunities to develop clinical skills, supportiveness, confidence and self-efficacy	Students: Medicine Rural (*n* = 44), metropolitan (*n* = 43)	Students rated the rural experience higher than the metropolitan experience across four areas: clinical teaching, opportunities to develop clinical skills, level of support in the clinical setting and self-reported confidence and self-efficacy	***
Ohta et al. (2019) Japan	Students’ perceptions of general medicine following community-based medical education in rural Japan	To determine medical students’ perception of general medicine including advantages and disadvantages of training in a community hospital	Validated pre- and post-placement achievement surveys 10 items on a 5-point Likert agreement scale Curriculum quality survey 12 items Semi-structured interviews	Students: Medicine (*n* = 15)	Increased knowledge of healthcare settings, models of health, communication with patients, and role clarification Curriculum content ratings indicated that the educational approach, content, and learning activities met the curriculum objectives Interview themes: driving forces for general medicine and roadblocks from multiple perspectives	***
Young et al. (2007) Australia	Knowing your allies: Medical education and interprofessional exposure	To evaluate and provide evidence of health profession and student development within an education program	Pre- and post-placement questionnaire (study-specific) 4-point Likert scale Research project task Open-ended questions (feedback)	Students: Medicine (*n* = 134) Health Professionals (*n* = 50) Disciplines: Child Health Nurse, Nurse Practitioner, Occupational Therapy, Pharmacy, Psychology, Physiotherapist, Podiatry, Registered Nurse, Speech Pathology, Social Work	80% of students reported positive changes in attitudes towards other professions Over 90% reported slight to moderate benefits for skill development 63% reported developing new skills	***
Young et al.(2008) Australia	Clinical location and student learning: Outcomes from the LCAP program in Queensland, Australia	To evaluate the learning and assessment provided in a rural generalist medical program for third-year medicine students	Mixed-methods case studies Questionnaire: Rural Rotation Survey (Parry et al.) Time 1—pre-placement, Time 2—two months, Time 3—post-placement Confidence of clinical competencies– core clinical competencies—70 procedures, rating: not at all confidence, confident with supervision, confident without Semi-structured interviews—post-placement: students, preceptors, spouses, community members	Students: Medicine (*n* = 3) LeichhardtCommunity Attachment Placement (LCAP) Tertiary hospital, (*n* = 6) Preceptors (*n* = 3)	LCAP students performed more named procedures than tertiary hospital counterparts and were more confident in procedural skills. Benefits include long-term contact with patients, opportunities for hands-on learning, and immersion in medical practice, with opportunities for informal and formal learning The benefits of collegial support were reported by the preceptors, and community members valued the student engagement and participation in social activities	***

*MMAT scoring: 100% quality criteria met = *****; 80% = ****; 60% = ***; 40% = **; 20% = *

**Table 5:** Characteristics of studies—quantitative study design

References	Title	Aims/objectives	Study design/methods Measures	Population	Key findings	MMAT Score
Aggar et al. (2020) Australia	Interprofessional primary healthcare student placements: Qualitative findings from a mixed-method evaluation	To investigate students’ attitudes, knowledge, skills and behaviours towards interprofessional collaboration and teamwork	Focus groups: Study-specific questions relating to interprofessional knowledge, attitudes and future practice	Students: Nursing (*n* = 32) Allied health (*n* = 30) Occupational Therapy, Speech Pathology, Podiatry	Students reported increased confidence and knowledge of interprofessional attitudes, scope of practice, communication skills and preparation for future practice	**
Daly et al. (2013) Australia	What factors in rural and remote extended clinical placements may contribute to preparedness for practice from the perspective of students and clinicians?	To identify the factors in an integrated, community-engaged rural placement that may contribute to preparedness for practice (P4P) from the perspective of students and clinicians	Semi-structured interviews (45-min) Questions not defined	Students: Medicine Three universities (*n* = 24) General practitioners (*n* = 8) Clinicians (*n* = 10)	Longitudinal rural placement contributed to PFP across clinical learning (e.g. procedural and clinical skills, service learning), personal and professional development (e.g., belonging, confidence and self-efficacy) and cultural skill development (e.g., cross-cultural care)	***
Furness et al. (2020) Australia	What students and new graduates perceive supports them to think, feel and act as a health professional in a rural setting	To explore the perspective of allied health students and new graduates on rural clinical placements’ role in contributing to thinking, feeling and acting like a health professional	Focus groups—study-specific questions on the impact of placement on students’ ability to think, feel, and act like health professionals	Students: Allied health students (*n* = 12) Physio, Occupational Therapy, Psychology, Registered Nurse, Speech Pathology, Social Work Graduates (*n* = 11)	Findings indicated that learning is supported through partnerships, opportunities for reflection, supervision and team connectedness. Factors to support students include quality learning, socialization and connectedness, independence and increased opportunities in the rural context	****
Gupta and Howden (2022) Scotland	Context and mechanisms of interprofessional learning during Longitudinal Integrated Clerkship	To research students’ perception of their learning during a rural longitudinal integrated clerkship and the transition back to a traditional hospital clerkship setting	Journalling—audio and written reflection Semi-structured interviews	Students: Medicine (*n* = 5)	Students reported that Primary Care settings promoted interprofessional learning and practices. Student learning was enhanced through inclusive teams, the absence of hierarchy, autonomy and authentic, contextualised learning environments	*****

*MMAT scoring:100% quality criteria met = *****; 80% = ****; 60% = ***; 40% = **; 20% = *

Over 60% of the studies included students from medicine (*n* = 11), and only three studies comprised multi-disciplinary student groups. Two studies included interprofessional education or interprofessional collaborative practice as a key focus for the placement design (Table 6). Of the studies that reported the placement setting (*n* = 14), over half were in Primary Care (*n* = 8) followed by rural hospitals (*n* = 4).

**Table 6:** Placement characteristics

		No. of studies	References
Disciplines	Allied health*	1	Furness et al. (2020)
	Medicine	11	Daly et al. (2013); Gupta and Howden (2022); Graham et al. (2023); Hunsaker et al. (2006); Lyon et al. (2008); McLean et al. (2010); Ohta et al. (2019); Rudland et al. (2011); Witney et al.(2018); Young et al. (2007); Young et al. (2008)
	Multi-disciplinary*	3	Aggar et al.(2020); McNair et al. (2005); Wakida et al. (2015)
	Nursing	3	Bennett et al. (2013); Edwards et al. (2004); Webster et al.(2010)
Placement setting	ACCHO*	1	Bennett et al. (2013)
	Hospital	4	Furness et al. (2020); Lyon et al. (2008); Ohta et al. (2019); Rudland et al. (2011)
	Multi-site	1	Daly et al. (2013);
	Primary care*	8	Aggar et al. (2020); Gupta and Howden (2022); Hunsaker et al. (2006); McNair et al. (2005); Wakida et al.(2015); Webster et al.(2010); Young et al.(2007); Young et al. (2008)
	Not defined	4	Edwards et al. (2004); Graham et al. (2023); McLean et al. (2010); Witney et al. (2018);
Placement length	2 weeks	2	McNair et al. (2005); Ohta et al. (2019);
	4—8 weeks	4	Bennett et al. (2013); Wakida et al.(2015); Webster et al.(2010); Young et al.(2007)
	16 weeks	2	Hunsaker et al. (2006); Lyon et al. (2008)
	12 months	4	Graham et al. (2023); Gupta and Howden (2022); Witney et al. (2018); Young et al.(2007)
	Mixed lengths	1	Daly et al. (2013)
	Not defined	5	Aggar et al. (2020); Edwards et al. (2004); Furness et al. (2020); McLean et al. (2010); Rudland et al. (2011)
Placement design	Inter-professional	2	Aggar et al. (2020); Young et al. (2007)
	Longitudinal integrated	4	Daly et al. (2013); Graham et al. (2023); Witney et al. (2018); Young et al.(2008)

^*^Allied Health: Health disciplines not included in the medical, dental or nursing professions. Primary care: General practitioner clinic, community clinic. ACCHO: Aboriginal Community Controlled Health Organization; Multi-disciplinary: Multiple health disciplines

### The impact of rural placement

Research question One was addressed by identifying skills, competencies, and key aspects of PFP reported in the included studies. The reported outcomes are collated in Tables 7 and 8. For example, the qualitative study by Daly et al., (2013) included semi-structured interviews; the themes reported in the findings were autonomy, confidence, professional identity, and references to clinical skills or competencies, including clinical procedural skills, cross-cultural competence, and patient education. A narrative discussion regarding each category in preparedness for practice (Table 7) and competency and skill development (Table 8) has been included below.

**Table 7:** Self-reported or self-assessed preparedness for practice

Article citation	Preparedness for practice				
	Autonomy	Confidence	Professional knowledge	Professional identity/belonging	Self-efficacy
Aggar et al.(2020)					
Bennett et al. (2013)					
Daly et al.(2013)	*	*		*	
Edwards et al.(2004)		*			
Furness et al.(2020)	*	*		*	
Graham et al.(2023)					
Gupta and Howden (2022)					
Hunsaker et al. (2006)		*	*		
Lyon et al.(2008)		*			*
McLean et al.(2010)					
McNair et al. (2005)		*			
Ohta et al.(2019)					
Rudland et al.(2011)					
Wakida et al.(2015)			*		
Webster et al	*	*			
Witney et al.(2018)					
Young et al.(2007)					
Young et al.(2008)			*		

Cross-cultural competencies: Indigenous health, cross-cultural communication, interaction and care; IPCP: Role clarification, patient-centred care, interprofessional attitudes and beliefs; Professional knowledge: Knowledge of healthcare systems

**Table 8:** Self-reported or self-assessed competency or skill

Article citation	Competency or skill								
	Clinical reasoning	Clinical/procedural skills	Communication	Complex cases	Cross-cultural competence	IPCP	Leadership	Patient education	Teamwork/Professional relationships
Aggar et al.(2020)			*			*			
Bennett et al. (2013)			*		*				
Daly et al.(2013)		*			*			*	
Edwards et al.(2004)									
Furness et al.(2020)									*
Graham et al.(2023)		*			*				
Gupta and Howden (2022)						*			
Hunsaker et al. (2006)									
Lyon et al.(2008)		*							*
McLean et al.(2010)		*							
McNair et al. (2005)						*			
Ohta et al.(2019)			*	*		*			
Rudland et al.(2011)		*						*	
Wakida et al.(2015)		*	*				*		
Webster et al					*				*
Witney et al.(2018)									
Young et al.(2007)						*			
Young et al.(2008)	*	*							*

Cross-cultural competencies: Indigenous health, cross-cultural communication, interaction and care; IPCP: Role clarification, patient-centred care, interprofessional attitudes and beliefs; Professional knowledge: Knowledge of healthcare systems

#### Preparedness for practice

Regarding PFP, increased autonomy, decision making and an ability to work independently were reported by students who had undertaken rural placements (Daly et al., 2013; Furness et al., 2020; Webster et al., 2010). As previously indicated, confidence (self-efficacy) is a common measure of progress in health education (Mullen et al., 2015). Not surprisingly, changes in self-assessed confidence were reported in 50% of the studies (*n* = 9). Improvements in confidence included interdisciplinary work (Aggar et al., 2020), interprofessional collaborative practice (McNair et al., 2005), cross-cultural care and Aboriginal health (Bennett et al., 2013). Students reported confidence and self-efficacy in recalling information and functioning in a team (Lyon et al., 2008), competence and organisation regarding placement attendance (Edwards et al., 2004) and overall confidence in clinical skills and competencies (Daly et al., 2013; Hunsaker et al., 2006; Young et al., 2008). Professional knowledge gained included professional issues and ethics, specialty options (Young et al., 2008), medical knowledge and population health (Wakida et al., 2015). Positive changes in professional belonging, professional identity and pre- to post-registration career transitions were reported (Daly et al., 2013; Furness et al., 2020).

#### Competency and skill development

Clinical and procedural skills were reported in 38% of studies (*n* = 7); however, the approach for assessing or measuring students’ perceptions regarding acquiring clinical or procedural skills varied across the studies. Studies comparing rural and urban learning environments reported rural students experienced improvements in clinical reasoning and confidence in procedural skills and clinical competencies compared to urban (metropolitan) students (Rudland et al., 2011; Young et al., 2008). Students attending both rural and urban placement settings indicated rural contexts provided greater opportunities to develop clinical skills (Lyon et al., 2008). Medicine students’ experiences across four universities indicated a significant level of agreement on skill development (McLean et al., 2010), and students from multiple disciplines and universities indicated clinical and procedural skills were a key aspect of learning during rural placement (Wakida et al., 2015).

Improved communication and interpersonal skills were reported concerning interprofessional collaborative practice (Aggar et al., 2020; Ohta et al., 2019). Students’ perceptions across broad domains indicated improvements in communication and leadership skills (Wakida et al., 2015), and the development of an empathic response to client/patient needs (Bennett et al., 2013). Patient education was identified as an area of improvement for students in rural settings (Daly et al., 2013) and when compared to urban contexts (Rudland et al., 2011). Cultural competence included gaining experience in cross-cultural care, client interactions, providing culturally appropriate care, and understanding the needs of Aboriginal and Torres Strait Islander clients (Bennett et al., 2013; Daly et al., 2013). Students felt that placement provided an opportunity to experience Aboriginal and Torres Strait Islander care and health issues (Graham et al., 2023; Webster et al., 2010).

Positive professional relationships with clinical supervisors, mentors, and peers were reported (Webster et al., 2010), in addition to confidence in their ability as team members (Lyon et al., 2008) and teamwork skills, including social support and connection with others (Furness et al., 2020). Positive changes in attitudes towards other professions and role clarification (interprofessional skills, knowledge and interprofessional collaborative practice—IPCP) were reported when interprofessional supervisory teams supported medicine students on placement (Young et al., 2008). Interprofessional student groups in Primary Care settings reported improved interprofessional knowledge and role clarification (Aggar et al., 2020). Similarly, an interprofessional learning placement model supported student competence in teamwork and interprofessional practice (McNair et al., 2005) and increased knowledge of complex cases (Ohta et al., 2019). The experience and perception of students transitioning from rural interprofessional clerkship to urban traditional clerkship were compared, and improvements in interprofessional knowledge on rural placement were indicated (Gupta & Howden, 2022).

#### Effective teaching and learning on placement—student perspective

As indicated above (Research Question One), the studies included in this review primarily reported students’ self-assessed confidence regarding PFP and competency and skill development. Research Question Two aimed to identify the competencies and skills health students believe are effectively taught, practised, and assessed in the rural environment. When charting the data, there needed to be clear references to assessment items or student feedback on assessment to address Research Question Two. Only one study reported that students were provided with a list of skills generated from the placement objectives, and students rated their experience. The detailed list of skills were not included in the article. However, the authors outlined 23 examination skills, 32 practical procedural skills, five investigation and interpretation skills, and 12 history-taking skills. The findings indicated that the students reported they did not experience 12% of the listed skills (Rudland et al., 2011). It was also reported that students experienced a lack of specialist training (McLean et al., 2010).

### Factors that influence learning

**Fig. 2:**
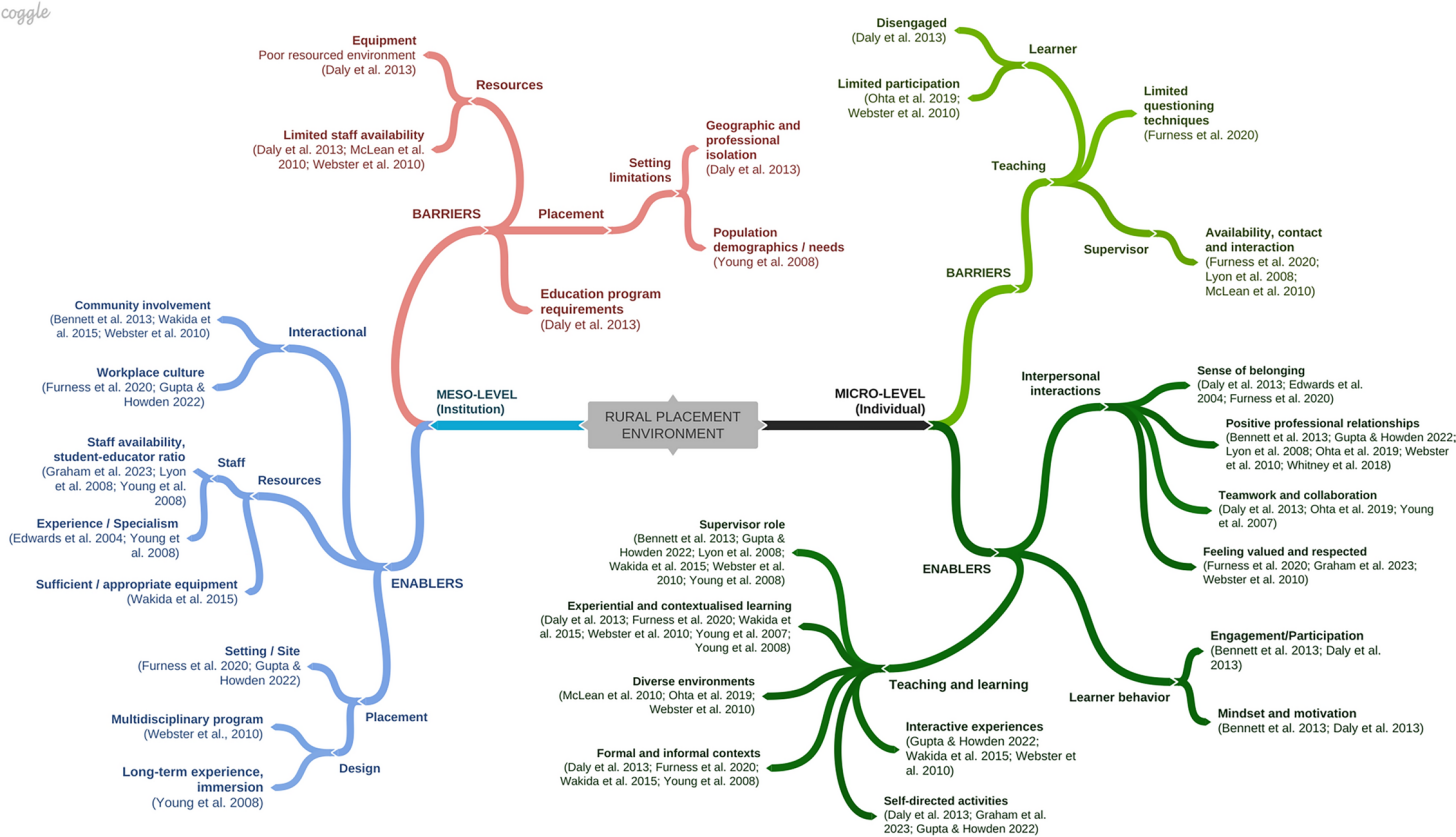
Reported enablers and barriers to learning in the rural learning environment

Data collection regarding enablers and barriers to learning was reported through focus group discussions and open-ended questionnaires where students shared their learning experiences. None of the included studies specifically used questioning to identify barriers and enablers for learning. To address Research Question Three, the extraction involved charting the reported learning experiences as factors operating at the levels present in Ecological Systems Theory (EST). The deductive analysis was framed by EST as recommended by Pollock et al. (2022). Half of the included studies (*n* = 9) included factors operating at both the micro and meso-level that enabled and supported learning. A summary of the themes and sub-themes relating to the factors identified in the included studies is provided in Fig. 2. For example, the barriers at the meso-level included ‘resources’ (theme) and ‘*limited staff availability* and ‘*equipment’* (sub-themes). The branches in the mind map (Fig. 2) include the themes and sub-themes. A narrative description of the categories for each sub-theme is included below.

At the institutional level (meso-level), the reported factors that created barriers to learning included the placement *setting* including limitations such as patient/client demographic and the reduced learning opportunities (e.g. few complex cases) (Young et al., 2008). Conversely, the enablers relating to placement included the placement *design* that supported learning through a multidisciplinary approach (Webster et al., 2010), and immersion in practising medicine through long-term experience and exposure to specialist teaching clinics (Young et al., 2008). In some cases, the placement *setting/site* (e.g. Primary Care) allowed students to observe multi-disciplinary collaboration (Gupta & Howden, 2022). Placement *setting limitations*, including geographic location, contributed to feelings of isolation and increased pressure to meet educational program requirements (Daly et al., 2013). Whereas interactional factors such as *community involvement* and engagement provided positive learning experiences enhanced by living in the community (Bennett et al., 2013; Wakida et al., 2015; Webster et al., 2010). Similarly, the importance of a welcoming *workplace culture* was highlighted (Furness et al., 2020), including clinical staff’s support of the placement program (Gupta & Howden, 2022). Resource limitations related to *staff availability*, contact and consistency (Daly et al., 2013; Lyon et al., 2008; McLean et al., 2010), amount of supervision (Furness et al., 2020), and specialty areas available (McLean et al., 2010), in addition to *equipment* limitations and a poorly resourced environment (Daly et al., 2013). In some instances, students reported that due to the complexity of the rural healthcare environment, rural supervisors were required to have extensive or *specialist experience* (Edwards et al., 2004; Graham et al., 2023; Lyon et al., 2008) and *staff availability* (staff resources) was indicated as an important positive factor on student learning (Graham et al., 2023; Lyon et al., 2008; Young et al., 2008).

Unsurprisingly, most individual (micro-level) factors were interpersonal or related to learner behaviour, and teaching and learning. In some instances, learner behaviour and participation and the role of the supervisor or mentor were highlighted. For instance, less proactive and *disengaged* students were reported as limiting their opportunities for formal and informal educational experiences (Young et al., 2008). On the other hand, *active participation* and student receptiveness positively influenced student learning (Daly et al., 2013).

Inadequate pre-placement *preparation* impacted student motivation (Ohta et al., 2019) and student knowledge gaps were also reported (Webster et al., 2010). Learning was positively influenced by student *mindset*, proactive behaviour (*motivation*), students actively seeking learning opportunities (e.g. home visits, health promotion activities) and becoming familiar with community nuances (Bennett et al., 2013). Social inclusion was linked to a sense of belonging (Daly et al., 2013) and feeling part of the clinical team (Edwards et al., 2004). Similarly, students shared the importance of socialisation and connectedness with peers, one’s profession, and other professions (Furness et al., 2020). Positive *professional relationships* extended to community members who shared their lived experiences (Ohta et al., 2019) and showed patience towards students (Bennett et al., 2013). Similarly, students could observe positive professional relationships through a reduced staff-student and interprofessional hierarchy (Young et al., 2007). In addition, enablers included nurturing clinical teams, resulting in trusting and supportive relationships (Gupta & Howden, 2022; Lyon et al., 2008). Students could engage in real-life projects involving collaboration among different health professionals (Young et al., 2008) and authentic teamwork and collaboration (Daly et al., 2013; Ohta et al., 2019). Finally, students reported feeling *valued* and *respected* as important factors (Furness et al., 2020; Graham et al., 2023; Webster et al., 2010).

The role of the supervisor was highlighted as limiting or creating a barrier to learning, for example, inadequate *questioning techniques* (Furness et al., 2020), minimal feedback and negative perceptions of students’ abilities (Lyon et al., 2008). Positive examples of the role of a supervisor involved modelling respect and professionalism, coaching, providing effective feedback, enthusiasm, and approachability (Bennett et al., 2013; Gupta & Howden, 2022; Lyon et al., 2008; Wakida et al., 2015; Webster et al., 2010). In addition, *self-directed* learning was enhanced by the student-supervisor relationship (Young et al., 2008). Self-directed learning activities increase a sense of responsibility (Daly et al., 2013; Graham et al., 2023) and autonomy (Gupta & Howden, 2022).

Finally, the rural context provided diverse learning environments for students (McLean et al., 2010; Ohta et al., 2019; Webster et al., 2010), including social interactions and *interactive experiences* (e.g. meetings and extra-curricular activities) (Gupta & Howden, 2022). Multiple experience-level student groups provided peer mentoring and discussion (Wakida et al., 2015) and peer interaction provided valuable social support (Webster et al., 2010). The rural context provided additional *informal (and formal)* learning experiences that students recognised as authentic learning opportunities (Daly et al., 2013; Furness et al., 2020; Wakida et al., 2015; Young et al., 2008).

## Discussion

Student experience on placement is crucial to professional identity and the professional self and influences future career pathways (Brownlee et al., 2015). Pre-professional socialization significantly impacts professional identity formation and future employability (Fitzgerald et al., 2024; Tomlinson & Jackson, 2021). This review aimed to contribute to the literature by exploring the reported findings from studies investigating the experiences and perspectives of pre-registration health students on rural placement regarding competency and skill development, as well as barriers and enablers for learning. The relatively small number of studies included in the review (*n* = 18) suggests a paucity of research specifically highlighting the students’ perspective regarding learning in the rural placement environment. Nonetheless, the findings have provided an overview of the reported competency, skill development and PFP. The results indicated that the studies predominantly focused on self-reported confidence regarding competency and skill development and a relatively small number of studies (*n* = 3) referred to autonomy, professional knowledge, belonging and self-efficacy. Similarly, complex cases, communication skills, clinical reasoning, leadership and patient education were identified as competencies or skills reported in a limited number of studies. The rural clinical environment provided valuable opportunities for health students through contextualised, authentic learning experiences however, the literature did not indicate that the student experience was highlighted or emphasised. The findings also highlighted a gap in the literature regarding students’ experiences and perspectives regarding the assessment and teaching of competencies and skills. Identifying the skills and competencies students believe they have successfully acquired in the rural learning contexts may, again, inform program design and provide insight into PFP. Identifying factors of influence *and* students’ perspectives may be beneficial for planning and developing future placement programs.

Confidence is often reported as a measure of self-efficacy. However, self-efficacy also relates to persistence and an individual’s ability to deal with challenges (Bandura, 1997) which can be supported through self-directed learning and increasing autonomy and agency. One could argue that pedagogy, teaching approaches, the learning environment and the interplay between these factors influence mastery of tasks and developing competence (and confidence).

The results also highlighted the lack of standardised measures for PFP and competency and skill development, which is unsurprising considering the lack of agreement in the literature regarding the conceptualisation of PFP (Aggarwal et al., 2023; Brennan et al., 2024; Monrouxe et al., 2017). Opportunities to work with and understand the health issues of Aboriginal and Torres Strait Islander Peoples were also identified as important. Previous studies have identified the transformative effects of undertaking placements with Aboriginal and Torres Strait Islander Peoples. A review by McDonald et al., (2018) reported that placements in rural areas that involved working with Aboriginal and Torres Strait Islander Peoples improved knowledge of racism, enhanced cultural awareness, improved knowledge of the complexity of cultural determinants of health, and increased interest in working with Aboriginal and Torres Strait Islander Peoples (McDonald et al., 2018).

The results also highlighted the reported enablers and barriers to learning on rural placement with reference to Ecological Systems Theory (EST; Bronfenbrenner, 1979), and this classification provides direction for designing and refining health education programs with effective rural placements. As outlined by Strasser and Neusy (2010) the complexity of the rural health contexts is well-documented in the literature dating back to the mid-1980s. Rural practitioners often work in isolation and shoulder higher clinical responsibilities and workload than urban practitioners. As a result, the rural health environment provides a rich and diverse learning experience for health students. The results reinforced the complexity of the placement learning environment and highlighted that some factors (e.g. the role of the supervisor) can be a double-edged sword creating both enablers and limitations for learning. There is a degree of bidirectional feedback between these factors, for example, student motivation, mindset and engagement influenced interpersonal relationships and learning activities that subsequently influenced the supervisor’s role, which in turn influenced the learning activities for the students.

As indicated in the results, community and relationships are central to rural learning, providing students with a safe place to learn culturally sensitive care and establish positive professional relationships (Purea et al., 2022). The literature included in this review highlighted the importance of factors operating at the micro (individual) level. It is also important to note that interpersonal factors interact with and are influenced by institutional factors (meso-level) and influence student experience and the learning environment. One particularly notable factor is professional relationships and a sense of connection to the professional team and, more broadly, the rural community. An important part of socialisation and professional identity formation is self-assessment, self-reflection and conscious and unconscious knowledge acquisition through experiential learning (Cruess et al., 2019). Forming connections and positive collaboration supports and influences the learner’s motivation and as a result, their performance and engagement in the learning environment (Deci et al., 2001). It is important to consider the student’s perspective regarding the enablers and barriers that influence this environment when planning and designing learning programs and placement experiences. Considering the learner’s motivation, their existing knowledge and learning needs align with the principles of andragogy and effective program development (Merriam & Bierema, 2013).

The findings from this review indicated a need for more research that explicitly highlights student voices regarding what does and does not support their learning. In particular, what are the skills and competencies they are not experiencing whilst on placement. Self-reported assessment of learning can provide feedback on the learning process however, learning is nuanced and complex and more detailed feedback from students may assist educators in refining health education.

### Implications for teaching and learning

As previously outlined, the United Nation’s Sustainability Development Goals specifically outline the importance of training, recruitment and retention of healthcare workers (Goal 3.C) (United Nations, 2015). The recruitment and retention of healthcare workers is pivotal in improving access the healthcare services and therefore, addresses one of the social determinants of health (WHO, 2024a, b). In order to adequately train (and retain) health students, they need to acquire more than skills and competencies alone; they must internalise their profession’s professional beliefs, values, and behaviours (Cruess et al., 2019). This process is influenced by role models, previous experience, and the ‘hidden curriculum’ in health education training (Bandini et al., 2017; Hafferty, 1998). Relatedness, a key aspect of intrinsic motivation, influences performance and learning (Deci et al., 2001) and the findings from this review highlight that feeling welcomed, valued, and respected are supportive factors in the rural environment. This was echoed in Hanna et al.’s (2021) systematic review of the barriers and facilitators for learning from the perspective of paramedic students. Hanna et al. indicated socialization and professional relationships were essential for student learning. This has been similarly detailed in a study exploring student perspectives regarding maximising connectivity in rural placements where medical students shared that they wanted connection across the rural, peer and clinical community which positively impacted further rural placement (Carrigan et al., 2024).

In addition to feeling like a health professional, acting like a health professional was an important consideration indicated by Hanna et al., (2021) for instance, autonomy and increased independence were facilitated in formal and informal learning experiences. This was similarly reported in the present review as well as the importance of self-directed learning. The findings reinforced how the learning environment (institutional factors) and the key stakeholders (interactional factors) influence student learning and engagement. Increasing autonomy and agency may be supported by providing health students with the opportunity to engage in authentic learning with long-term contact with clients, supporting students to engage in decision-making and work independently (Daly et al., 2013; Young et al., 2008). Providing health students with an understanding of why self-directed learning supports and enhances their experience on placement may prove valuable when designing health education programs.

In addition to the previously noted lack of measures for PFP, there was limited reference to professional standards and competencies. For instance, the health students were not asked to refer to professional standards of competencies when providing feedback or completing study measures. It could be suggested that students may benefit from self-assessment with reference to their health discipline’s professional standards. Similarly, there was only one study (i.e., Furness et al., 2020) that reported what students perceived supported them to think, feel and act like a health professional (i.e. competence, relatedness and autonomy).

Adequately trained supervisors and mentors were identified as influential for student learning, and this is reiterated by a recent study exploring nursing students’ perception of preceptor competence (Alhassan et al., 2024). The study by Alhassan et al., however, presents the student perception ofpreceptor competence, and not students’ perception of *their* ability to develop and acquire skills on placement (as was the focus of this review). Based on our findings, we could argue that interpersonal communication and skills training for supervisors, as well as skill development regarding self-directed tasks, are important for increasing not only relatedness but autonomy and, subsequently, competence (and confidence).

One final consideration is the interdependent nature of the factors operating at the macro-level within the professional system (e.g. clinical placement setting) and the educational system (e.g. university or institution) (D’Amore & Oandasan, 2005). The findings indicated that shared goals, vision and workplace culture (meso-level factors) were influential factors in supporting health students’ learning, and these factors are influenced by effective leadership, governance and institutional practices. The example of shared goals and workplace culture is related to the sense of belonging, feeling respected and valued as an active team member (Furness et al., 2020; Graham et al., 2023). It could be suggested that capturing the student experience and perspective could benefit leaders as well as educators across both systems. In addition, linking the student feedback to professional competencies and standards may prove beneficial for program development.

### Future research

The findings of this review suggest several areas that warrant further investigation. The majority of research focused on the experience of medicine students, thus identifying opportunities for exploration of the student perspective of other health disciplines. Similarly, even though the search did not have any geographic limitations, the majority of studies were from Australia, indicating a gap in the literature. This could suggest the potential for international collaboration to explore the enablers and barriers to learning and different pedagogical approaches across different rural communities in multiple countries. As noted, developing a measure of PFP may provide consistency for future research in the field. Finally, we may also consider how we can assess or determine what supports health students to think, feel and act like rural health professionals.

### Limitations

As healthcare competencies encompass a broad range of skills across disciplines, some research may be absent from this review due to the selective searching process. This review aimed not to capture all articles relating to competency development but to map the peer-reviewed literature that highlights student perspectives and experiences regarding the skills and competencies effectively taught in the rural learning environment. In addition, we aimed to provide an understanding of the reported enablers and barriers to learning from the perspective of health students. A grey literature was not completed due to the author’s decision to include only peer-reviewed, empirical studies. Non-peer reviewed sources were not included therefore creating a limitation of the review. The identified studies included predominantly medicine students, resulting in an underrepresentation from other health disciplines. Similarly, most of the identified studies were conducted in Australia, which presents a limitation regarding the representation of other countries.

## Conclusion

This scoping review revealed pre-registration health students’ perspective of rural placements, especially concerning competency and skill development, enablers and barriers to learning. The review drew from diverse studies, including mixed-methods, quantitative and qualitative approaches and mapped the reported competency and skill development on rural placement. We found that student experience on placement is crucial to professional identity formation and influences future career pathways. The relatively small number of studies in this review indicates that this area warrants further attention and investigation. Similarly, few studies explicitly captured health students’ perspectives regarding PFP and competencies and skills that were not acquired during their rural placement experience.

Due to the nature of the rural and remote environment, community and relationships were found to be central to learning on rural placements. The review reiterated the importance of interpersonal interactions for student learning and skill development and serves as a timely reminder of the barriers to learning experienced by students in the professional environment and supports the argument that we need to further explore the experience of students in order to support the future health professionals. Program development can be informed and enhanced by student experience, perspective and student-identified strategies and approaches that support understanding and engagement. Ensuring the health of rural communities relies on effective health education programs and effective placement design to adequately prepare health students for practice as dedicated rural health professionals.

## Supplementary Information

Below is the link to the electronic supplementary material.

Supplementary file1 (DOCX 19 kb)

## Data Availability

Data is available upon request from the corresponding author.
