# Supplementary material for: Preparedness for practice, competency and skill development and learning in rural and remote clinical placements: A scoping review of the perspective and experience of health students
Source: Adv Health Sci Educ Theory Pract. 2024 Sep 30;30(3):935–72. doi: 10.1007/s10459-024-10378-4 (PMC12119662; doi:10.1007/s10459-024-10378-4)
Supplement: Supplementary file 1 — Supplementary file1 (DOCX 19 kb) [file 10459_2024_10378_MOESM1_ESM.docx]

**Supplementary material:**

| **Database** | **Search strategy** | **Results** |
| --- | --- | --- |
| Informit | Title ("preparedness" OR "readiness" OR "education" OR "training" OR "development" OR "professional competence" OR "clinical competence" OR "competency-based education" OR "clinical skill" OR "comptenc*" OR "student perception" OR "student perspective" OR "student experience") AND ("health care student*" OR "health professions student" OR "nursing student" OR "medical student" OR "occupational therapy student" OR "allied health student" OR "physical therapy student" OR "pre-registration" OR "pre-licensure" OR "nursing trainee" OR "health student") AND ("rural" OR "remote" OR "rural community" OR "remote community") AND ("clinical placement" OR "clinical clerkship" OR "preceptorship" OR "work experience" OR "placement" OR "in-service training" OR "medical education" OR "clinical school" OR "community-based medical education")  OR AB ("preparedness" OR "readiness" OR "education" OR "training" OR "development" OR "professional competence" OR "clinical competence" OR "competency-based education" OR "clinical skill" OR "comptenc*" OR "student perception" OR "student perspective" OR "student experience") AND ("health care student*" OR "health professions student" OR "nursing student" OR "medical student" OR "occupational therapy student" OR "allied health student" OR "physical therapy student" OR "pre-registration" OR "pre-licensure" OR "nursing trainee" OR "health student") AND ("rural" OR "remote" OR "rural community" OR "remote community") AND ("clinical placement" OR "clinical clerkship" OR "preceptorship" OR "work experience" OR "placement" OR "in-service training" OR "medical education" OR "clinical school" OR "community-based medical education") | 21 |
| Scopus | TITLE-ABS ( ( "preparedness"  OR  "readiness"  OR  "education"  OR  "training"  OR  "development"  OR  "professional competence"  OR  "clinical competence"  OR  "competency-based education"  OR  "clinical skill"  OR  "comptenc*"  OR  "student perception"  OR  "student perspective"  OR  "student experience" )  AND  ( "health care student*"  OR  "health professions student"  OR  "nursing student"  OR  "medical student"  OR  "occupational therapy student"  OR  "allied health student"  OR  "physical therapy student"  OR  "pre-registration"  OR  "pre-licensure"  OR  "nursing trainee"  OR  "health student" )  AND  ( "rural"  OR  "remote"  OR  "rural community"  OR  "remote community" )  AND  ( "clinical placement"  OR  "clinical clerkship"  OR  "preceptorship"  OR  "work experience"  OR  "placement"  OR  "in-service training"  OR  "medical education"  OR  "clinical school"  OR  "community-based medical education" ) ) | 690 |
| Ebsco host  Academic Search Complete, Applied Science & Technology Source, Business Source Complete, CINAHL Complete, EconLit, Education Source, GreenFILE, Health Business Elite, Health Source - Consumer Edition, Health Source: Nursing/Academic Edition, Humanities Source, Library, Information Science & Technology Abstracts, MAS Ultra - School Edition, MasterFILE Complete, Newspaper Source Plus, Newswires, Psychology and Behavioral Sciences Collection, Regional Business News, Religion and Philosophy Collection, SPORTDiscus with Full Text, The Serials Directory, MasterFILE Premier Reference eBook Subscription (EBSCOhost), MAS Reference eBook Collection, MLA Directory of Periodicals, MLA International Bibliography with Full Text | S8 TI "preparedness" OR "readiness" OR "education" OR "training" OR "development" OR "professional competence" OR "clinical competence" OR "competency-based education" OR "clinical skill" OR "comptenc*" OR "student perception" OR "student perspective" OR "student experience"  S9 AB "preparedness" OR "readiness" OR "education" OR "training" OR "development" OR "professional competence" OR "clinical competence" OR "competency-based education" OR "clinical skill" OR "comptenc*" OR "student perception" OR "student perspective" OR "student experience"  S10- S8 OR S9  S11 TI "health care student*" OR "health professions student" OR "nursing student" OR "medical student" OR "occupational therapy student" OR "allied health student" OR "physical therapy student" OR "pre-registration" OR "pre-licensure" OR "nursing trainee" OR "health student"  AB "health care student*" OR "health professions student" OR "nursing student" OR "medical student" OR "occupational therapy student" OR "allied health student" OR "physical therapy student" OR "pre-registration" OR "pre-licensure" OR "nursing trainee" OR "health student"  S13- S11 OR S12  S14- TI "rural" OR "remote" OR "rural community" OR "remote community"  S15- AB "rural" OR "remote" OR "rural community" OR "remote community"  S16- S14 OR S15  S18- TI "clinical placement" OR "clinical clerkship" OR "preceptorship" OR "work experience" OR "placement" OR "in-service training" OR "medical education" OR "clinical school" OR "community-based medical education"  S19- AB "clinical placement" OR "clinical clerkship" OR "preceptorship" OR "work experience" OR "placement" OR "in-service training" OR "medical education" OR "clinical school" OR "community-based medical education"  S20- S18 OR S19    S10 AND S13 AND S16 AND S20 | 79 |
| Embase | S1‘preparedness' OR 'readiness' OR 'education' OR 'training' OR 'development' OR 'professional competence' OR 'clinical competence' OR 'competency-based education' OR 'clinical skill' OR 'comptenc*' OR 'student perception' OR 'student perspective' OR 'student experience':ab,ti  S2- 'health care student*' OR 'health professions student' OR 'nursing student' OR 'medical student' OR 'occupational therapy student' OR 'allied health student' OR 'physical therapy student' OR 'pre-registration' OR 'pre-licensure' OR 'nursing trainee' OR 'health student':ab,ti  S3- 'rural' OR 'remote' OR 'rural community' OR 'remote community':ab,ti  S4- 'clinical placement' OR 'clinical clerkship' OR 'preceptorship' OR 'work experience':ab,ti    S5- #1 AND #2 AND #3 AND #4 | 154 |
| Web of science | (TI=(("preparedness" OR "readiness" OR "education" OR "training" OR "development" OR "professional competence" OR "clinical competence" OR "competency-based education" OR "clinical skill" OR "comptenc*" OR "student perception" OR "student perspective" OR "student experience") AND ("health care student*" OR "health professions student" OR "nursing student" OR "medical student" OR "occupational therapy student" OR "allied health student" OR "physical therapy student" OR "pre-registration" OR "pre-licensure" OR "nursing trainee" OR "health student") AND ("rural" OR "remote" OR "rural community" OR "remote community") AND ("clinical placement" OR "clinical clerkship" OR "preceptorship" OR "work experience" OR "placement" OR "in-service training" OR "medical education" OR "clinical school" OR "community-based medical education"))) OR AB=(("preparedness" OR "readiness" OR "education" OR "training" OR "development" OR "professional competence" OR "clinical competence" OR "competency-based education" OR "clinical skill" OR "comptenc*" OR "student perception" OR "student perspective" OR "student experience") AND ("health care student*" OR "health professions student" OR "nursing student" OR "medical student" OR "occupational therapy student" OR "allied health student" OR "physical therapy student" OR "pre-registration" OR "pre-licensure" OR "nursing trainee" OR "health student") AND ("rural" OR "remote" OR "rural community" OR "remote community") AND ("clinical placement" OR "clinical clerkship" OR "preceptorship" OR "work experience" OR "placement" OR "in-service training" OR "medical education" OR "clinical school" OR "community-based medical education")) | 69 |
| Medline | S#1- (((TI=("preparedness" OR "readiness" OR "education" OR "training" OR "development" OR "professional competence" OR "clinical competence" OR "competency-based education" OR "clinical skill" OR "comptenc*" OR "student perception" OR "student perspective" OR "student experience" )) AND TI=("health care student*" OR "health professions student" OR "nursing student" OR "medical student" OR "occupational therapy student" OR "allied health student" OR "physical therapy student" OR "pre-registration" OR "pre-licensure" OR "nursing trainee" OR "health student" )) AND TI=("rural" OR "remote" OR "rural community" OR "remote community" )) AND TI=("clinical placement" OR "clinical clerkship" OR "preceptorship" OR "work experience" OR "placement" OR "in-service training" OR "medical education" OR "clinical school" OR "community-based medical education" )  S#2- (((AB=("preparedness" OR "readiness" OR "education" OR "training" OR "development" OR "professional competence" OR "clinical competence" OR "competency-based education" OR "clinical skill" OR "comptenc*" OR "student perception" OR "student perspective" OR "student experience" )) AND AB=("health care student*" OR "health professions student" OR "nursing student" OR "medical student" OR "occupational therapy student" OR "allied health student" OR "physical therapy student" OR "pre-registration" OR "pre-licensure" OR "nursing trainee" OR "health student" )) AND AB=("rural" OR "remote" OR "rural community" OR "remote community" )) AND AB=("clinical placement" OR "clinical clerkship" OR "preceptorship" OR "work experience" OR "placement" OR "in-service training" OR "medical education" OR "clinical school" OR "community-based medical education" )  S#1 or S#2 | 88 |
| Psychifo | S1- [title("preparedness" OR "readiness" OR "education" OR "training" OR "development" OR "professional competence" OR "clinical competence" OR "competency-based education" OR "clinical skill" OR "comptenc*" OR "student perception" OR "student perspective" OR "student experience" ) AND title("health care student*" OR "health professions student" OR "nursing student" OR "medical student" OR "occupational therapy student" OR "allied health student" OR "physical therapy student" OR "pre-registration" OR "pre-licensure" OR "nursing trainee" OR "health student" ) AND title("rural" OR "remote" OR "rural community" OR "remote community" ) AND title("clinical placement" OR "clinical clerkship" OR "preceptorship" OR "work experience" OR "placement" OR "in-service training" OR "medical education" OR "clinical school" OR "community-based medical education" )](https://www.proquest.com/recentsearches.recentsearchtabview.recentsearchesgridview.scrolledrecentsearchlist.checkdbssearchlink:rerunsearch/C384E76D058C4214PQ/None/$N?t:ac=RecentSearches)  S2- [abstract("preparedness" OR "readiness" OR "education" OR "training" OR "development" OR "professional competence" OR "clinical competence" OR "competency-based education" OR "clinical skill" OR "comptenc*" OR "student perception" OR "student perspective" OR "student experience") AND abstract("health care student*" OR "health professions student" OR "nursing student" OR "medical student" OR "occupational therapy student" OR "allied health student" OR "physical therapy student" OR "pre-registration" OR "pre-licensure" OR "nursing trainee" OR "health student") AND abstract("rural" OR "remote" OR "rural community" OR "remote community") AND abstract("clinical placement" OR "clinical clerkship" OR "preceptorship" OR "work experience" OR "placement" OR "in-service training" OR "medical education" OR "clinical school" OR "community-based medical education")](https://www.proquest.com/recentsearches.recentsearchtabview.recentsearchesgridview.scrolledrecentsearchlist.checkdbssearchlink:rerunsearch/AB72A6F3AFF14DFBPQ/None/$N?t:ac=RecentSearches)  S1 OR S2 | 85 |
